# Determinants of Satisfaction With Health Services by Dimensions of Care: An Age-Stratified Analysis

**DOI:** 10.1093/geroni/igaa057.555

**Published:** 2020-12-16

**Authors:** Helen-Maria Vasiliadis, Isabelle Pitrou, Djamal Berbiche

**Affiliations:** 1 University of Sherbrooke, Sherbrooke, Quebec, Canada; 2 University of Sherbrooke, Longueuil, Quebec, Canada; 3 universite de Sherbrooke, Sherbrooke, Quebec, Canada

## Abstract

Studies that examined satisfaction with care in older adults are scarce. The aim of this research was to analyse satisfaction among older adults considering mental health, socio-clinical and health system factors and by age category. Data come from the Étude sur la Santé des Aînés Services study including 1,624 adults aged ≥65 years recruited between 2011-2013 in primary care in Quebec. Patient satisfaction was assessed during interviews with questions adapted from the Primary Care Assessment Survey. Mental health (anxiety, depression, suicidal ideation, psychological distress, cognition), social support, quality of life, the presence of pain and chronic conditions were self-reported. Health service use was extracted from administrative registries. Logistic regressions stratified by age were used to examine the associations of low satisfaction in three dimensions of care. For continuity of care, the determinants of low satisfaction were pain and attraction index for psychiatric services in adults 65 to 75 years versus anxiety, cognition and hospitalizations in adults 75 years and older. For patient-provider interactions, the determinants were psychological distress, attraction index for psychiatric services in adults 65 to 75 years versus quality of life and cognition in adults 75 years and older. For adequacy of care, anxiety, psychological distress, social support, pain, quality of life and attraction index for psychiatric services were significant in adults 65 to 75 years versus quality of life and cognition in adults 75 years and older. Results highlight different patterns of satisfaction by age category that should be used to improve care delivered in primary care.

